# Barriers to the management of children under five exposed to HIV in the rural areas of South Africa

**DOI:** 10.4102/curationis.v44i1.2073

**Published:** 2021-03-08

**Authors:** Sibusiso F. Buthelezi, Regis R.M. Modeste, Deliwe R. Phetlhu

**Affiliations:** 1 Department of Health Sciences Education, University of Cape Town, Cape Town, South Africa; 2 Department of Nursing Science, Cape Peninsula University of Technology, Cape Town, South Africa; 3 Department of Nursing Science, University of the Western Cape, Cape Town, South Africa

**Keywords:** barriers, management, HIV-exposed children, children under five, rural areas, South Africa

## Abstract

**Background:**

South Africa has made enormous progress in reducing mother-to-child transmission (MTCT) of human immunodeficiency virus (HIV), however, MTCT and AIDS related death persist among children particularly in the rural areas. Lack of adherence to health policies and guidelines implementation remain one of the contributory factors to poor management of HIV-exposed children. Hence, the need to deeply explore the complexity of the problems and understand the barriers to the management of HIV exposed children in the rural areas.

**Objectives:**

To explore and synthesise the barriers to the management of children under 5 years old exposed to HIV in rural areas in South Africa.

**Method:**

An integrative literature review was conducted. An electronic search was conducted on several databases. The researchers applied the Boolean ‘ AND’/‘OR’ in combination with phrases such as ‘HIV infection*’, ‘HIV transmission’, ‘HIV-exposed infant*, child*, and neonate*’ and ‘South Africa*’. Included studies were limited to South Africa, and articles were written in English and published in peer-reviewed journals from 2005 to 2018. Both qualitative and quantitative studies between 2005 and 2018 were utilised.

**Results:**

The findings highlighted that healthcare institution-related barriers, healthcare provider-related barriers, patient-related barriers and Socio-economic-related barriers were the significant barriers to the management of HIV-exposed children in the rural areas.

**Conclusion:**

Continuous engagement with all relevant stakeholders should remain a priority in protecting HIV-exposed children. It is evident that there exist gaps in the current implementation of prevention of mother-to-child transmission (PMTCT), especially in rural areas. Therefore, intervention strategies that could improve implementation of PMTCT policy guidelines for HIV-exposed children in rural areas are needed.

## Introduction

It is indisputable that South Africa (SA) has made significant progress in reducing the child mortality rate through the programme for human immunodeficiency virus (HIV) prevention of mother-to-child transmission (PMTCT). The children under-five mortality rate has declined from 80.1 child deaths per 1000 live births to 45.0 child deaths per 1000 live births between 2002 and 2018 (Stats SA 2018). Children under five refers to children from 0 to exactly 5 years of age, whereas the under-five mortality rate is the probability (expressed as a rate per 1000 live births) of a child in a specified year dying before reaching the age of 5 years (United Nations [UN] 2016).

Even though the reduction in child mortality has been hailed as a success, the reality of individual provinces is not represented. Predominantly, rural and urban provinces need to be highlighted as that will shed light and facilitate an equal spread of resources or intentionally directed interventions.

South Africa consists of nine provinces, wherein five (Eastern Cape [EC], KwaZulu-Natal [KZN], Limpopo, Mpumalanga and North West [NW]) have more than 40% of their population living in rural areas (Atkinson 2014). This affects health outcomes, such as mother-to-child transmission (MTCT). In the NW province, which is a predominantly rural province, the infant HIV polymerase chain reaction (PCR) test was positive around 10 weeks and was reported at 1.1% in 2018, which is higher than the national target of 0.9%. The Northern Cape (NC) province was at 1.4%, and EC province was at 1.2% compared to 0.5% in the Western Cape (WC), which is predominantly an urban province (Massyn, Pillay & Padarath 2018). This evidence shows that the progress in eliminating MTCT in rural areas is plodding.

It is unquestionable that SA has performed well in reducing MTCT and child mortality. The PMTCT programme has been the leading intervention over the past 15 years, which has been used to curb the MTCT and child mortality. The PMTCT has been continuously updated over the years. This is from Option A (meaning that HIV-positive mothers are eligible for antiretroviral therapy [ART] during pregnancy and intrapartum period to minimise the chances of MTCT) and Option B (meaning that mothers are eligible for triple ART until they stop breastfeeding, given that they do not qualify for lifelong ART). Lastly, Option B+ (meaning that regardless of the CD4 cell count all pregnant mothers in the PMTCT programme are commenced on a lifelong ART) (Fords, Crowley & Van der Merwe 2017). South Africa implemented Option B+ in 2015 (Goga et al. 2018), and it is the latest update made in the PMTCT policy guidelines. The changes include initiating lifelong ART immediately after making a diagnosis on all women in the PMTCT programme, infant HIV PCR testing at birth, as well as increased accessibility to PMTCT. However, MTCT persists in South African health facilities, especially during post-partum (Akinsanya et al. 2017).

Prevention of mother-to-child transmission services starting from antenatal care (ANC) to the delivery period have been well delivered with few exceptions, such as late bookings, unknown HIV status and mothers not on antiretroviral (ARV) treatment. The very few exceptions that still exist must not be allowed to continue and reverse the gains made so far. However, the most critical period to manage HIV-exposed children appears to be the postnatal period. Obtaining the results of HIV PCR test on time, at 10 weeks and 18 months, HIV testings for children are some of the crucial moments of managing HIV-exposed children under 5 years (National Department of Health [NDoH] 2014). Massyn et al. (2018) reported PMTCT performance to be below the national target in rural areas. For example, the mothers’ postnatal visit as early as within 6 days in the district of Ngaka Modiri Molema (NMM) was reported at 63.6%, and in Buffalo City in the EC, it was found to be at 43.2%. Note that both are below the national target of 70.9% (Massyn et al. 2018). The HIV testing around 18 months for HIV-exposed children remains low in rural areas, with records of the district of NMM in the NW province being at 37.8%. While the NW is documented at 58.5%, with EC being recorded at 64%, all these are lower than the national target of 78.9% (Massyn et al. 2018). This unsatisfactory performance reported in the rural areas is a result of multiple challenges that continue to affect multiple stages of the PMTCT programme. These are clinics being far away from the place of living, lack of transport, shortage of material resources, human resources, poverty, financial constraints, strong cultural beliefs or practices and inaccessible geographical location because of bad untarred roads (Doherty et al. 2009; Mafune, Lebese & Nemathaga 2017; Meehan et al. 2015).

Generally, MTCT has drastically declined in SA as it was reported at 0.9% decrease from 1.3% in 2016/2017 (Massyn et al. 2018). As a result, many children under five have been saved from contracting HIV and dying of Acquired Immunodeficiency Syndrome (AIDS) related conditions before they even turn 5 years (UN 2016). Despite this general decline in the country, the same cannot be said in the inter-provincial and inter-district areas with regard to MTCT. In NMM in NW, the MTCT rate was reported at 1.0%; in Dr Ruth Segomotsi Mompati in NW, it was at 1.6%; in Joe Gqabi in EC, it was at 1.9%; and in John Taolo Gaetsewe in NC, it was at 3.6%. These are all above the national target of 0.9% (Massyn et al. 2018).

Children under 5 years, mainly in rural areas, continue to die from preventable diseases (UN 2016). The implication is that although the fight of MTCT has progressed far much better in urban areas (such as WC), the rural areas seem to be left behind (such as in NW and EC). It is undeniable that MTCT continues to happen because some of the HIV-exposed children test positive at 10 weeks. Therefore, it is concerning that the majority of HIV-exposed children are not brought back to the clinic for confirmatory test at 18 months (Massyn et al. 2018) as per the PMTCT guidelines. The poor testing of children exposed to HIV at 18 months cannot be taken lightly; hence, the barriers and reasons that result in mothers and/or caregivers not bringing back their children to the clinic, which has severe implications for children under 5 years who are exposed to HIV, need to be well understood. This needs to be understood in its context, which is part of what this integrative literature review aims to do. The women and/or caregivers’ behaviour of not bringing a child for confirmatory tests at 18 months and mothers who stop taking ARVs because their health has improved tremendously (Tshililo et al. 2019) continue to put the lives of HIV-exposed children under five at risk of dying from AIDS-related illnesses (such as pneumonia) even before they turn 5 years of age. The evidence shows that the highest child mortality rates are in rural areas (Massyn et al. 2018). The slow progress reported is concerning because it undermines the gains made to curb MTCT so far. On the other hand, it shows that there are still leakages in the cascade of PMTCT (Kendall et al. 2015) which cannot be ignored in order to reach the elimination of MTCT (EMTCT).

Therefore, without accelerating the speed of progress, the majority of children under five who are living in the rural areas have no chance to see the sustainable development goals (SDGs) target of an HIV-free generation in 2030 (UN 2016). To adequately respond to the SDG motto of ‘leaving no one behind’ (UN 2016:21) and SDG 3, which says ‘ensure healthy lives and promote well-being for all at all ages’ (UN 2016:6) the focus of EMTCT should not only be directed at urban areas and those areas that are easily accessible, but should also be directed at remote rural areas. These remote areas need to be given as much attention as possible. It is imperative to improve the PMTCT with contextuality-based intervention strategies to reach the end of MTCT in 2030.

Despite the continuous upgrade performed in the PMTCT programme, it seems that children under five exposed to HIV are not fully benefiting from the strengthened PMTCT programme. The majority of children who test positive because of MTCT remain a serious threat to the PMTCT programme while undermining the vision to end EMTCT by 2030.

It is evident that PMTCT could not be implemented as a one-size-fits-all programme; it needs to be shaped to respond to and tackle challenges for a specific context, such as the rural areas. Doing so may assist in closing the existing gaps in the implementation of the PMTCT programme. Therefore, effective intervention strategies to improve implementation of PMTCT in rural areas in SA are needed. Hence, the purpose of this review is to explore deeply and understand the barriers to the management of children under 5 years, which still exists in rural areas in SA. Doing so could provide insights into the development of intervention strategies that are contextually based and appropriate to improve the implementation of PMTCT programmes in rural areas in SA.

## Aims of the review

The aim of this integrative literature review was to explore and synthesise the barriers to management of children under 5 years exposed to HIV in rural areas in SA.

## Methods

An integrative review method was considered appropriate to fully comprehend the barriers that contribute to poor management of HIV-exposed children in rural areas, which was the aim of this review. In addition, this method was chosen because of its strength to combine qualitative and quantitative research studies in order to genuinely comprehend the phenomenon under review (Whittemore & Knafl 2005). However, conducting a review that combines qualitative and quantitative studies is complex and requires authors to pay serious attention to the details (Torraco 2016). The stages of the integrative literature review design, as stated by Whittemore and Knafl (2005), which include problem identification, literature search, data evaluation, data analysis and presentation, were followed. This integrative review was guided by the following question: what are the barriers that exist with regard to the management of HIV-exposed children under five in the rural areas in SA?

### Literature search

A comprehensive electronic search was conducted on the following databases: PubMed; Medline; EBSCOhost: Africa-wide information, Cumulative Index to Nursing and Allied Health Literature (CINAHL) and Health Source: Nursing/Academia Edition; and Google Scholar. The researchers applied the Boolean ‘AND’/‘OR’ in a combination of phrases such as ‘HIV infection*’; ‘HIV transmission’; ‘HIV-exposed infant*’, child*, neonate*’; ‘PMTCT, management of HIV in babies*’; ‘Rural area*’ and ‘South Africa*’. Medical Subject Headings (MeSH) was used to solicit more terms in the database vocabularies. The ancestry searching was also used as an additional search strategy by reviewing the reference lists of reviewed articles (Torraco 2016).

**FIGURE 1:**
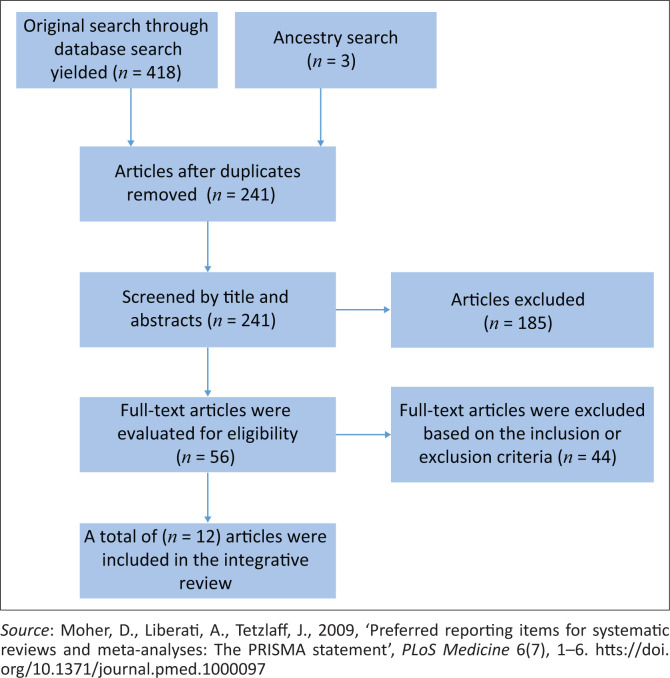
Flow chart of search strategy.

A flow chart strategy (Figure 1) was used to depict the process and the final number of selected articles. A total of 12 articles (Figure 1) were included in the review that met the inclusion criteria.

### Inclusion and exclusion criteria

The inclusion criteria were primary studies published in peer-reviewed journals between 2005 and 2018 in English language. The 2005 date was selected because the PMTCT programme was officially implemented in 2002 in SA (Barron et al. 2013), and in 2004 ARV treatment was made available (Sprague, Chersich & Black 2011). Additionally, the authors assumed that health facilities and individuals who are involved in the implementation of this PMTCT policy and its guidelines would have adapted well after 3 years of its official inception.

Studies focusing on PMTCT in the rural areas and conducted in SA were included. Both qualitative and quantitative studies were included. Articles were excluded if studies were not conducted in rural areas, SA, and focus was not on the PMTCT programme. Unpublished dissertations, thesis, conference proceedings and reports (grey literature) were purposefully excluded as the focus was on peer-reviewed published articles.

### Data evaluation

Each of the final included articles was analysed independently by two reviewers. As stated by Whittemore and Knafl (2005:549), there is ‘no gold standard tool for evaluating quality in the integrative review; the process depends on the sampling frame’. Thus, we adapted two critical appraisal checklist tools to appraise both qualitative and quantitative studies. All qualitative studies were critically appraised using the Qualitative Assessment and Review Instrument (QARI) critical appraisal tool (Pearson 2004) as illustrated in Table 2. All quantitative studies were appraised using Bowling’s (2009) checklist, as shown in Table 3.

The QARI critical appraisal tool for qualitative studies has 10 questions, whereas Bowling’s checklist for quantitative studies has 20 questions to appraise the rigour of included studies.

These critical appraisal tools provide reviewers with a long detailed checklist instead of a numerical scoring system to critically evaluate the studies on the quality of the methodology in order to make an informed judgement on the final included articles. The authors felt that studies should meet 11–16 items on the checklist to qualify to be included in the review. The qualitative studies had to meet seven and above the items on the checklist to be deemed suitable for inclusion. Some of the items on the checklist were adapted from Pitt et al. (2012).

### Data analysis

Given that integrative literature reviews’ method of analysis remains poorly developed, researchers must use their creativity during the analysis process (Whittemore & Knafl 2005). In this review, the authors used a qualitative content analysis method to answer the review question. The qualitative data analysis method has been found to be appropriate to conduct analysis for the integrative review (Sandelowski 2000). The authors extracted information from the quantitative articles in meaningful units in order to create clear interpretation. This process is called qualitising, which refers to a process where ‘quantitative data are transformed into qualitative data’ (Sandelowski, Voils & Barroso (2006:8). All included articles were read multiple times and coded when patterns were identified. Then codes were compared and merged to create themes and subthemes as presented in Table 4. This exercise was performed by the three authors independently. During the analysis process, a matrix was used to display the data in order to identify patterns and to better perform comparison across all included studies. All authors met to discuss the final themes and subthemes to achieve consensus on the end results.

### Ethical considerations

This article followed all ethical standards for research without direct contact with human or animal subjects.

## Results

### Study demographics

A total of 12 articles that met the inclusion criteria were included in the review. This review was deliberately limited to SA; thus, all included studies were conducted in SA. The majority of studies were qualitative studies (*n* = 8), followed by quantitative studies (*n* = 4). All 12 included studies were conducted in rural areas: (NW, *n* = 3; Mpumalanga, *n* = 2; KZN, *n* = 1; Limpopo, *n* = 3; and EC, *n* = 3. The selection process of all included studies is presented in the flow chart of the search strategy in Figure 1, whereas the summary of all the studies included is presented in Table 1. Finally, the qualitative appraisal checklist is presented in Table 2, and the quantitative appraisal checklist is given in Table 3.

**TABLE 1:** Characteristics and summary of articles included in the review.

Author	Setting	Sample or methods	Research design	Findings
Mlambo et al. (2018)	Mpumalanga (Nkangala district)	Sixty-six participants were purposively selected. They consisted of HIV-positive pregnant and postnatal women, grandmothers and healthcare providers. Semi-structured in-depth interview and focus group discussion were conducted. Thematic inductive analysis was conducted.	Qualitative exploratory design	Women who were pregnant for the second time were presenting very late (6 months) for ANC avoiding multiple clinic visits. Long distance to the clinic, fear of rude nurses and fear to do HIV test were the reasons for late clinic visits.
Kaswa et al. (2018)	Eastern Cape (Mbekweni Health Centre, King Sabata Dalindyebo sub-district)	Twenty pregnant women were purposively selected. Semi-structured in-depth interviews and focus group interviews were conducted. Thematic analysis was conducted.	Qualitative study	Reasons highlighted for late clinic visits included long waiting times, women were scared to test for HIV and did not have money for transport. Fear of losing their partners after being tested positive prevented some women from attending the clinic.
Wilford et al. (2018)	KwaZulu-Natal (Ugu, Umgungundlovu, and Harry Gwala districts)	Fifteen community healthcare workers and 30 women (10 were pregnant and 20 had already delivered) were all purposively selected. In-depth interviews were conducted. Thematic content analysis conducted.	Qualitative exploratory design	Healthcare workers had inadequate knowledge regarding PMTCT protocols. Training of healthcare workers on PMTCT protocols and examination of mother and infant. Lack of tools – that is, thermometers, MUAC tapes and how to utilise such tools for household visits – was a challenge.
Hanrahan and Williams (2017)	Limpopo (Polokwane district)	Twenty-one registered nurses were purposively selected. Semi-structured interviews were conducted. Thematic analysis was undertaken.	Qualitative, descriptive design	There were staff shortages and medication shortages in clinics. Healthcare workers needed refresher training on PMTCT policies and guidelines. Late bookings for ANC, lack of family support to HIV-positive women and usage of traditional remedies were reported.
Habedi et al. (2016)	North West (Madibeng sub-district)	Ten HIV-positive pregnant women wer purposively selected. Semi-structured interviews and thematic analysis were conducted.	Qualitative, exploratory, descriptive contextual design	Staff shortages and long waiting times were reported. Women were told to come back on the next day without being checked, and HIV-positive women were frustrated by that.
Sithole and Khunou (2016)	North West (Ngaka Modiri Molema district)	Nine midwives were purposively selected. Semi-structured face-to-face individual interviews and thematic analysis were conducted.	Qualitative, exploratory, descriptive design	Women were reported to attend the clinic after 28 weeks for the first time. Nurses’ negative attitude contributed to late clinic visits. Some midwives were not knowledgeable about PMTCT. Antenatal care services were provided by one midwife.
Useh et al. (2013)	North West (Mafikeng)	Three hundred twenty pregnant women were selected through convenience sampling. Self-constructed questionnaire was used for data collection. SPSS and simple descriptive statistics were undertaken for analysis.	Descriptive and cross-sectional study	Some pregnant women were lacking knowledge about MTCT. Out of 175 pregnant women, 49.1% were able to explain MTCT, whereas 33.5% did not know what MTCT is, and 7.4% were undecided.
Ebonwu et al. (2018)	Limpopo (Capricorn district)	Eight hundred ten pregnant women participated in the study from Capricorn and Tlokwe sub-districts. Questionnaire and face-to-face interviews were used to collect data. STATA 13 was undertaken during the analysis.	Cross-sectional study	Women were consulting traditional healers first before coming to the clinic. Long distance to clinic, long waiting times and infrequent transport were highlighted as barriers to access the healthcare institutions.
Peltzer et al. (2016)	Mpumalanga (Nkangala district)	One hundred five participants (managers, nurses, lay counsellors and PMTCT and mother-to-mother counsellors) participated. A 23-item questionnaire on PMTCT protocol was completed by CHC staff. An audio computer-assisted survey instrument that was administered using headphones was completed by all the participants. A descriptive statistics was undertaken for analysis.	Clinical trial	There was a lack of human resources, lack of adherence to PMTCT protocol in the clinics. Lack of PMTCT for infant delivery and ART commencement were noted. Healthcare workers needed refresher training to accurately implement PMTCT protocols.
Ajewole et al. (2013)	Limpopo (maternity ward of a provincial hospital)	One hundred sixty-nine postpartum women participated in the study. A questionnaire was used to collect data. Descriptive statistics was performed. Data were analysed using the Epi Info Software version 3.4.1 (2007).	Cross-sectional descriptive study	Pregnant women were scared to know their HIV status because of social stigma. The women who tested HIV-positive were enrolled in the PMTCT. However, one woman tested HIV-positive but did not enrol in the PMTCT programme.
Skinner et al. (2005)	Eastern Cape (Qaukeni Local Municipality)	Twenty-nine participants were purposively selected: 13 individual interviews and 26 focus group discussions were held. Atlas.ti was used for the analysis.	Qualitative study	Staff shortages, clinics run out of medicine, long distance and long waiting times were highlighted as barriers to accessing clinics and benefiting from PMTCT. Women had lack of support from their partners and families. Fear to test for HIV because of stigma was reported. Pregnant women were using traditional medicine.
Adeniyi et al. (2015)	Eastern Cape (King Sabata Dalindyebo Municipality)	Twenty-four mothers of HIV-exposed infants were purposively selected. Semi-structured interviews and thematic content analysis were conducted.	Qualitative study	Women were scared to disclose their HIV status, fearing rejection and stigmatisation from families and the community. Others did not want their babies to be tested for the very same reason. Some women did not bring their children for the 6 weeks HIV PCR test.

ANC, antenatal care; MUAC, mid-upper arm circumference; SPSS, statistical package for the social sciences; STATA, statistical analysis; HIV, human immunodeficiency virus; PCR, polymerase chain reaction; PMTCT, prevention of mother-to-child transmission; MTCT, mother-to-child transmission.

**TABLE 2:** Qualitative studies critical appraisal checklist.

Criteria	Yes	No
1. Congruity between stated philosophical perspective and research methodology	7	1
2. Congruity between methodology and research question or objective	8	0
3. Congruity between methodology and methods used to collect data	8	0
4. Congruity between methodology and representation and analysis of data	8	0
5. Congruity between methodology and interpretation of results	8	0
6. There is a statement locating the researcher culturally or theoretically	1	7
7. The influence of the researcher on the research and vice versa is addressed	0	8
8. Participants and other voices are adequately represented	8	0
9. Ethical according to current criteria, evidence of ethical approval	7	1
10. Conclusions drawn flow from analysis or interpretation of data	8	0

*Source:* Pearson, A., 2004, `Balancing the evidence: Incorporating the synthesis of qualitative data into systematic reviews’, *JBI Reports* 2(2), 45–64. https://doi.org/10.1111/j.1479-6988.2004.00008.x

**TABLE 3:** Quantitative studies critical appraisal checklist.

Criteria	Yes	No
1. Aims and objectives clearly stated	4	0
2. Hypothesis or research question clearly specified	2	0
3. Dependent and independent variables clearly stated	3	1
4. Variables adequately operationalised	4	0
5. Design adequately described	4	0
6. Method appropriate	4	0
7. Instrument used tested for reliability and validity	3	1
8. Sample, inclusion or exclusion and response rate described	4	0
9. Statistical errors discussed	4	0
10. Ethical consideration	3	1
11. Was the study piloted?	3	1
12. Statistical analysis appropriate	4	0
13. Results reported and clear	4	0
14. Results reported related to hypothesis and literature	4	0
15. Limitations reported	3	1
16. Conclusions do not go beyond limit of data and results	4	0
17. Findings able to be generalised	1	3
18. Implications discussed	4	0
19. Conflict of interest with sponsor	1	3
20. Data available for scrutiny and re-analysis	1	3

*Source*: Bowling, A., 2009, *Research methods in health: Investigating health and health services*, Open University Press, Maidenhead.

### Study findings

The analysis of all 12 reviewed studies yielded four main themes on the barriers to management of HIV-exposed children in rural areas in SA, namely, health system-related barriers, patient-related barriers, healthcare provider-related barriers and socio-economic-related barriers. The four main themes and subthemes are presented in Table 4.

**TABLE 4:** Identified Themes and subthemes.

Themes	Subthemes
1. Healthcare institution-related barriers	Shortage of staff Shortage of HIV test kits and ARVs Long waiting periods at the clinic Long distance to the clinic
2. Healthcare provider-related barriers	Lack of knowledge about PMTCT Healthcare workers’ negative attitude
3. Patient-related barriers	Late booking Stigma Cultural beliefs
4. Socio-economic-related barriers	Transport costs Lack of financial support from partner or family

HIV, human immunodeficiency virus; ARV, antiretrovirals; PMTCT, prevention of mother-to-child transmission.

## Healthcare institution-related barriers

In this review, several healthcare institution-related barriers to the management of HIV-exposed children under five and living in rural areas in SA were identified. These are the barriers that reflect the reality of what happens at the healthcare institutions. They include staff shortage and long waiting periods in the clinic, amongst others.

### Shortages of staff

The shortage of staff was identified in six articles included in this review (Habedi, Nolte & Temane 2016; Hanrahan & Williams 2017; Kaswa, Rupesinghe & Longo-Mbenza 2018; Peltzer et al. 2016; Sithole & Khunou 2016; Skinner et al. 2005). Shortage of staff in clinics was highlighted as one of the contributing barriers that hinder the management of HIV-exposed children. This review found that in some of the clinics, there was only one midwife providing assistance in the ANC to all women who seek these services (Kaswa et al. 2018; Sithole & Khunou 2016). At times when clinics are jam-packed, nurses would instruct some of the women to go back home and come back the following day because they were not coping with the workload (Habedi et al. 2016; Hanrahan & Williams 2017). The PMTCT service was introduced to clinics without any additional staff to clinics that are already understaffed (Peltzer et al. 2016; Skinner et al. 2005), creating more challenges for the provision of PMTCT services. The shortage of staff including highly skilled trained staff such as nurse-initiated management of antiviral therapy (NIMART) in primary healthcare level in the rural areas robbed the majority of people, including women and their children, from receiving high-quality care. Without adequate healthcare workers at the primary level, the progress to successfully manage HIV-exposed children would remain unsatisfactory.

### Shortage of HIV test kits and antiretrovirals

The shortage of HIV test kits, as well as ARVs for mothers, was identified in two articles included in this review (Hanrahan & Williams 2017; Skinner et al. 2005). Clinic attendance remains very important during ANC and beyond to ensure that women and their children’s lives are kept healthy by taking ARVs for themselves and to PMTC during pregnancy and postnatal care. In this review, the researchers tand HIV test kits (Hanrahan & Williams 2017; Skinner et al. 2005). Furthermore, this review highlighted that nurses agreed that the challenging barrier in their clinic was shortage of ARVs and they further reported being frustrated by this barrier of running out of ARVs because they had to drive and get the ARVs and other supplies such as HIV test kits from the hospital by themselves (Hanrahan & Williams 2017). The shortages of ARVs and having to drive to fetch ARVs and HIV test kits from other hospitals or clinics have a high likelihood to contribute to the long waiting period experienced by women and caregivers in the clinics. Additionally, it may contribute to the loss to follow-up because of women and/or caregivers being sent back home with their children because of stock-out. These barriers show that more work needs to be performed in a faster pace to achieve Ideal Clinic realisation of having adequate medicine and supplies in the clinics, particularly in the rural areas. Many set targets (90-90-90 2020, National Development Plan [NDP] 2030, SDGs 2030) will not be achieved if the situation does not change at the primary healthcare level.

### Long waiting period at the clinic

The long waiting periods were identified in three articles included in this review (Habedi et al. 2016; Hanrahan & Williams 2017; Kaswa et al. 2018). These articles showed that women are keen to attend clinics but oftentimes are discouraged by the long queues and the long time they spend at the clinic (Kaswa et al. 2018). In this review, it was found that women complained that they sit and wait for a long time before they are attended to by a healthcare worker. This is despite the fact that they arrive at the clinic as early as 06:00 in the morning, but they would go home after 13:00 in the afternoon (Habedi et al. 2016; Hanrahan & Williams 2017). In one of the studies (Habedi et al. 2016) included in this review, it was noted that women living with HIV were frustrated to be sent back home without any help. In another article, it was highlighted that pregnant women (36 weeks) were complaining that it is difficult for them to sit on the queue for a very long time, even though they left their homes very early in the morning (Kaswa et al. 2018). The long waiting time in the clinics noted in the included reviews was not the only barrier that women and/or caregivers were unhappy about, but also the long distance that they had to travel to the clinic. The long waiting period in the clinics contradicts the vision of an Ideal Clinic model where patients will not spend more than 3 h, as per National Department of Health (NDoH) benchmark, despite the services they come for. This is because patients who come to the clinic for maternal and child health services spend way more than 3 h at the clinic (Egbujie et al. 2018). The implication of long waiting period at the clinics could result in women and/or caregivers and their children not being attended to by a healthcare worker on that day because of the work overload that the healthcare workers are faced with. Given the barriers that mothers and/or caregivers have to overcome, chances of not going back to the clinic on the next day are very high, resulting in the loss to follow-up, and missed opportunity for early infant diagnosis (EID).

### Long distance to the clinic

The long distance to the clinic was identified in four articles (Ebonwu et al. 2018; Habedi et al. 2016; Mlambo et al. 2018; Skinner et al. 2005). Even though pregnant women are encouraged to book ANC as early as possible, but living very far from the clinic contributed to the missed opportunities of the PMTCT (Habedi et al. 2016). One article showed that pregnant women opted to come to the clinic at a very later stage (36 weeks of pregnancy) because they get tired of travelling a long distance to the clinic (Mlambo et al. 2018). Rural area clinics are unjustifiably far away from where people live. This has a severe negative effect particularly when it comes to the management of HIV-exposed children under 5 years. Women, especially at a later stage of their pregnancy, end up not going to the clinic at all, especially if they do not have money. They cannot even attempt to begin to walk. In other articles, the long distance to the clinic that women and/or caregivers have to travel remains one of the significant barriers to access healthcare services (Ebonwu et al. 2018; Kaswa et al. 2018; Skinner et al. 2005). Hence, the focus must be redirected to rural areas to significantly improve access to healthcare facilities the same way as it is in the urban areas (Egbujie et al. 2018).

## Healthcare provider-related barriers

In this review, several healthcare provider-related barriers to the management of HIV-exposed children under five and living in rural areas in SA were identified. These barriers are significant to rural healthcare professionals because they have a severe potential to continue to compromise the quality of care expected to be rendered to women and their children and make the environment unwelcoming. These barriers include lack of knowledge regarding PMTCT guidelines and healthcare workers’ negative attitude.

### Lack of knowledge about prevention of mother-to-child transmission

The lack of knowledge was identified in five articles (Hanrahan & Williams 2017; Peltzer et al. 2016; Sithole & Khunou 2016; Useh et al. 2013; Wilford et al. 2018). It is imperative that healthcare workers and women living with HIV remain knowledgeable and well updated with the PMTCT services, as this is the instrument to manage HIV-exposed children. The gap in knowledge regarding MTCT and training was identified in both healthcare workers and pregnant women. One article reported that out of 175 pregnant women, 86 could explain MTCT, 76 did not know what MTCT is and only 13 were undecided (Useh et al. 2013).

Some of the healthcare workers openly acknowledged that their knowledge of MTCT was inadequate. Similarly, in one of the studies included in this review noted:

I do have knowledge, but it is not adequate, perhaps I need to be given additional information, there are questions that they (mothers) ask where you find that I will not be confident when I respond to them. (Wilford et al. 2018:5)

The lack of knowledge was also noted amongst midwives, with the mention of their lack of knowledge about PMTCT (Sithole & Khunou 2016). This highlights the need for extra training, as noted that healthcare workers reiterated that ‘everybody should be trained so that we do not say somebody is not around or she/he is ill’ (Skinner et al. 2005:119). There is a need for an adequate number of highly skilled trained healthcare providers such as NIMART in the rural areas to mitigate the situation whereby women and their children are sent back home when a particular staff member is sick or has a family emergency. Over and above, highly skilled healthcare workers are needed at the primary healthcare level in order to provide high-standard quality care.

### Healthcare workers’ attitude

The healthcare workers’ negative attitude was identified in three articles (Kaswa et al. 2018; Mlambo et al. 2018; Sithole & Khunou 2016). The authors noted that there was a negative attitude of healthcare workers, and this served as one of the contributing factors that hinder the uptake of PMTCT services (Mlambo et al. 2018). The participants were unhappy about how healthcare workers treat them when attending the clinic (Sithole & Khunou 2016). One participant highlighted that she did not like her clinic because ‘a clinic nurse turned her away, and the nurse told her to come back when she feels the baby moving’, and the participants were told that, ‘you are not paying here; it’s free, if you are in a rush go for medical aid’ (Kaswa et al. 2018:3). This negative attitude of healthcare workers resulted in participants having to attend clinics where nurses do not shout at them, even though such clinics were far from their place of living (Kaswa et al. 2018; Sithole & Khunou 2016).

On the other hand, there were some positive experiences with healthcare workers. Some of the participants agreed that ‘nurses are good here (a particular clinic) but they are so busy, always helping’ (Kaswa et al. 2018). With such a situation, it becomes a vicious cycle that negatively impacts the management of HIV-exposed children under 5 years. This is because the clinics where the staff attitude is positive end up being overcrowded, which, in turn, increases the patients’ waiting period, as well as the distance to be travelled to the clinic, as the pregnant women and mothers start avoiding their nearest clinics because of the ill-treatment they receive from healthcare workers. One way or another, the attitude of healthcare workers contributes very robustly to the barriers of managing the HIV-exposed children under 5 years. It is a behaviour that cannot be taken lightly in order to achieve EMTCT.

## Patient-related barriers

In this integrative literature review, a number of patient-related barriers to the management of HIV-exposed children under 5 years and living in rural areas in SA have been identified. These barriers include late bookings, stigma and cultural beliefs.

### Late bookings

Five of the 12 articles reviewed reported that pregnant women were attending ANC very late than the recommended period of less than 20 weeks (Ebonwu et al. 2018; Hanrahan & Williams 2017; Kaswa et al. 2018; Mlambo et al. 2018; Sithole & Khunou 2016). In this review, it was found that healthcare workers highlighted late booking as one of the barriers that result in pregnant women missing opportunities in the PMTCT programme (Sithole & Khunou 2016). Some of the participants would come to the clinic after 28 weeks; others would come when they are about to deliver their babies (Kaswa et al. 2018; Sithole & Khunou 2016). At times, women come early for bookings, but they are told by the healthcare staff to come back at a later stage (Ebonwu et al. 2018).

Missed opportunities that are disadvantageous to a mother and baby were noticed when other pregnant women admitted that for their third pregnancy, they came to the clinic when they were 31–35 weeks pregnant (Hanrahan & Williams 2017; Mlambo et al. 2018). This postponement results in delays in starting treatment planned for the PMTCT programme. For instance, it was noted that a woman presented to the clinic at 8 months pregnancy, and that was when she found out for the first time that she was HIV-positive (Mlambo et al. 2018; Sithole & Khunou 2016). Different reasons for late booking were noted. For example, some women were of the view that 36–40 weeks is too long; therefore, it is decided that they would book later at 31–35 weeks (Kaswa et al. 2018). Others were discouraged by the negative behaviour of staff members (Mlambo et al. 2018) and the fact that clinic was too far from their place of living (Hanrahan & Williams 2017; Kaswa et al. 2018). In this regard, it is challenging to implement the PMTCT programme as required by the guidelines and alternately that affects the health of the infants or under-five babies. Therefore, the country still reports high rates of new HIV infections, increased mortality rates and/or comorbid disorders associated with HIV.

### Stigma

Six of the 12 articles reviewed reported that the majority of pregnant women were afraid to take the HIV test and others were not willing to disclose their HIV status (Adeniyi et al. 2015; Ajewole, Sparks & Omele 2013; Kaswa et al. 2018; Sithole & Khunou 2016; Skinner et al. 2005; Wilford et al. 2018). This review found that 66.7% of women were afraid to discover their HIV status and very much concerned about social stigma (Ajewole et al. 2013). In addition, women said that they preferred not to know their HIV status because of the potential of being found to be HIV-positive (Kaswa et al. 2018; Skinner et al. 2005). Some of the participants would deny the results after being tested HIV-positive (Sithole & Khunou 2016). The fear of being tested prevented pregnant women, not all, from attending ANC because they knew that in order to benefit from the PMTCT programme, they must be tested.

The fear of knowing their own HIV status would even result in women deciding to come to the clinic in the last month of their pregnancy to avoid being tested for HIV (Mlambo et al. 2018). In other instances, some women admitted knowing their HIV-positive status but were afraid to disclose to their boyfriends to avoid being rejected and accused of infidelity (Adeniyi et al. 2015; Kaswa et al. 2018). Failure to disclose one’s HIV status to an immediate family or partner remains a serious barrier in managing HIV-exposed children, especially in instances where the mother is away for a long time and leaves a child with her family which is a common phenomenon, particularly in the rural areas. Thus, it results in situations whereby the child is not given ART as prescribed because no one will know that they should give the child medication as required. This results in the non-adherence to the PMTCT programme. Again, continuous education about HIV and AIDS in rural areas must be strengthened to empower people with knowledge of HIV and AIDS to neutralise the level of stigma that is directed at those living with HIV. In addition, healthcare workers must also refrain from the stigmatisation attitude towards women living with HIV when seeking services in the healthcare facilities. Such attitude has a serious potential of discouraging women from testing for HIV and/or even attending the healthcare facility, which overall puts their life at risk and that of the child to contract HIV.

### Cultural beliefs

The cultural beliefs were identified in six articles as a barrier to the management of HIV-exposed children under five (Ebonwu et al. 2018; Hanrahan & Williams 2017; Kaswa et al. 2018; Mlambo et al. 2018; Sithole & Khunou 2016; Skinner et al. 2005). These articles showed that some women are still influenced by their cultural beliefs on how to deal with their pregnancy (Mlambo et al. 2018). In one of the articles, it was noted that women culturally believe that once they become pregnant, they should stay at home, and not go to public spaces which include clinics (Sithole & Khunou 2016). Some cultures still hold a belief that once a woman becomes pregnant, she should not be visible in public. The reason behind such a belief is that an unborn child may catch evil spirits through their mother; hence, public spaces are forbidden. Rural areas remain one of the areas where traditional practices are still adhered to in modern SA. In addition, women continue to take traditional medicine while pregnant and attend the clinic at the same time (Hanrahan & Williams 2017). At times, the pressure comes from the elders in the family; their pregnant women are advised to drink traditional medicine to cleanse their blood and protect the unborn child (Mlambo et al. 2018). At times, women book later in the clinic because they first seek care from traditional healers.

Furthermore, this review found that once pregnancy was confirmed, 35% of women admitted that they seek care from traditional healers (Ebonwu et al. 2018). Other participants agreed to have used traditional remedies together with ART (Kaswa et al. 2018). The act of consulting traditional healers has the potential to contribute to late ANC booking, which disrupts the management of HIV-exposed children under 5 years. Similarly, the continuation of taking traditional remedies in conjunction with ART has to be discouraged at all levels. Continuous education about implications of prioritising traditional doctors when pregnant and mixing traditional medicine with ARVs must be revitalised.

## Socio-economic-related barriers

In this review, a number of socio-economic-related barriers to the management of HIV-exposed children under five and living in rural areas in SA have been identified. These barriers reflect the reality of poor socio-economic conditions that people live under in the rural areas and difficulties in accessing the healthcare institutions. These barriers include transport costs and transport availability, and lack of support from the partner and/or family members.

### Transport cost and availability

The transport concerns were identified in four articles (Ebonwu et al. 2018; Kaswa et al. 2018; Mlambo et al. 2018; Skinner et al. 2005). These articles showed that transport challenges are not only about the costs but also about the availability of transport and the type of transport that can access the area where people live despite the condition of the roads. Participants reported that at times they did not go to the clinic because they did not have money, and they could not afford to use a private taxi that costs about R250 (Kaswa et al. 2018). A rural area is often linked to scarce transport opportunities. For example, there were reports of participants having money for transport and being prepared to go to clinic, but the scarcity of transport in the rural areas resulted in them not attending the clinic. Therefore, accessing the services in place for the management of HIV-exposed children who are under 5 years of age (Ebonwu et al. 2018; Mlambo et al. 2018) and in the village where the quality of roads is poor and underdeveloped (Sewell & Desai 2016) remains a hindering factor for women to access clinics and benefit from the PMTCT services. This was noted from the review, with reports of pregnant women dying because of unavailability of cars, and a spelt-out need for cars as a mode of transport to clinics (Skinner et al. 2005). These barriers point out the need to strengthen and fast tract the integrated management of childhood illnesses (IMCI) in rural healthcare facilities. Strengthening the primary healthcare services at district and sub-district levels is crucial and requires urgent intervention. The set targets outlined in the NDP (2030:51) state that ‘health system should provide quality to all at the point of services’. In addition, the SDGs that state ‘leaving no one behind and ensure healthy lives and promote well-being for all at all ages’ may not be achieved if the focus is not redirected to rural healthcare in a faster pace than it is now to ensure unhindered access to healthcare facilities by women and their children.

### Lack of financial support from partner or family

The lack of financial support was another barrier identified in four articles (Ebonwu et al. 2018; Hanrahan & Williams 2017; Kaswa et al. 2018; Skinner et al. 2005). These articles revealed that women were reluctant to disclose that they are pregnant, especially the ones who were not married because of being scared to be left by a boyfriend, and therefore, lose financial support. The majority of women in rural areas depend on their partners financially; hence, the fear of being left by their partners was a concern to them. Without money for transport to attend clinic appointments as per PMTCT guidelines, it would have dire consequences on their HIV-exposed children in the ANC, delivery and postnatal period. The disappearance of these men left the women without any financial support to take care of the child not only to afford transport costs but also to afford food for the child. It is concerning that partners and families when they reprimand the loved ones they do that by cutting them off from financial support. It would have been a different story if men leave their partners but continue to take full responsibility for the child, especially the HIV-exposed children.

In this review, the researchers found that women alluded that once men find out that they are pregnant, the next day they disappear (Kaswa et al. 2018). In this review, it was noted that few women (7%) were supported and accompanied by their partner to a clinic, whereas 30% of women expressed a negative emotion, sadness and were scared for being alone upon confirming pregnancy (Ebonwu et al. 2018). This review noted that most women reported having very little financial support from families and partners. In addition, this review found that women without strong support around them tend to book later for ANC services (Hanrahan & Williams 2017; Skinner et al. 2005), which makes it challenging to manage a child that is exposed to HIV from contracting a vertical transmission.

## Discussion

The aim of this integrative literature review was to explore and synthesise the barriers to poor management of children exposed to HIV in the rural areas in SA. The four main themes identified (see Table 4) connected to healthcare institution-related barriers, healthcare provider-related barriers, patient-related barriers and socio-economic barriers. The significant findings of this integrative literature review show that rural areas continue to be faced with various bottlenecks that hinder women and children from benefiting from the services offered by healthcare institutions, such as accessing and remaining on the PMTCT programme for the management of HIV-exposed children under 5 years of age.

The rural healthcare institutions are mandated to provide PMTCT services and all other health services to the rural population. Elimination of MTCT relies on the healthcare institution’s day-to-day operations. All pregnant women are expected to benefit from the PMTCT services despite their location, whether they are in urban or rural areas. However, this review revealed the various barriers to the management of HIV-exposed children. For example, when it comes to the execution of these services, nothing is performed about the staff ratio, leading to staff shortage. Instead, the expectations are that institutions must provide PMTCT services without fail, yet they are understaffed and under-resourced.

The shortage of staff in rural healthcare facilities remains one of the significant barriers for women not to fully access ANC services and postnatal services. These are services provided in combination with the PMTCT services aimed at managing HIV-exposed children under 5 years of age.

This staff shortage has a ripple effect. Pregnant women are encouraged to come to the clinic as early as possible so that they benefit from the ANC services, including early enrolment in the PMTCT programme if needed. However, because of the shortage of staff, some of the women are told to go back to their homes and come back on the following day (Cataldo et al. 2017). This is a clear indication that the existing staff members are not coping with the work overload; hence, women complain of waiting for longer times before being attended to (Frost, Jenkins & Emmink 2017; Rodriguez et al. 2017). This review found that women also expressed their frustration of waiting long times at the clinics (Yah & Tambo 2018). It cannot be right that one staff member provides the entire ANC services to a large group of pregnant women (Kaswa et al. 2018). In the rural areas, to achieve EMTCT in a faster pace, services such as maternal & child health, integrated management of childhood illness (IMCI) and family planning services must be well integrated into primary healthcare. Thus, each healthcare facility should have an adequate number of well-trained health professionals (Tshililo et al. 2019); otherwise, women and their children in the rural areas will be left behind, which would have devastating implications for SA when it comes to ending new infections and the AIDS epidemic by 2030.

Moreover, men must support their partners during the pregnancy period, not only emotionally, physically, psychologically and spiritually, but also financially. The behaviour that continues to be practised by other men, which is to cut off financial support once the relationship has ended, has to cease because some women depend on their men for finances to travel to the clinic. Men need to be strongly educated about the implications caused by their failure to take responsibility for supporting a child even though the relationship with the mother has ended.

The other barrier noted in this review is that healthcare workers lack knowledge regarding PMTCT guidelines because these guidelines are not static; they are continuously updated. In this review, some of the healthcare workers openly acknowledged that their knowledge of MTCT was inadequate (Wilford et al. 2018). Previous researchers (Akal & Afework 2018; Fondoh & Mom 2017; Nkomo et al. 2015; Suryavanshi et al. 2018) have highlighted that there is a lack of training of healthcare providers on PMTCT guidelines. It is therefore imperative that healthcare workers are continuously trained to stay abreast of relevant information for best practices in managing HIV-exposed children under 5 years old. This would ensure that all healthcare staff are knowledgeable and therefore would provide adequate services to women living with HIV and HIV-exposed children. To have well-trained health professionals who will provide quality healthcare especially at the primary healthcare level is one of the NDP objectives which should be achieved not later than 2030. Thus, the focus should be channelled to rural healthcare by ensuring that every healthcare facility has more than one NIMART-trained staff. It cannot be right that the entire ANC services are delivered by one person. When a person is ill or has to attend to family emergencies, women who seek PMTCT services should not be turned back because no one else is trained to provide such services.

It is essential that all pregnant women are tested for HIV and those who test HIV-positive should be initiated on ART immediately as suggested in the PMTCT guidelines, Option B+. However, the challenges that were found in this review are that clinics in the rural areas are still running out of HIV testing kits and medication. Nurses at times have to drive to other hospitals to ask for medication and other medical supplies. These findings concurred with studies conducted by Yah and Tambo (2018), Suryavanshi et al. (2018), Cataldo et al. (2017), Fondoh and Mom (2017) and Rodriguez et al. (2017) that there was a stock-out of ART, Nevirapine (NVP), shortage of HIV test kits both for HIV and pregnancy. The shortage of ARVs and HIV test kits in the clinics undermine not only the 90-90-90 targets but also the achievements gained through the PMTCT programme so far and a potential to make the EMTCT a reality in SA.

There should be a well-organised effective supply chain management to avoid this ongoing barrier, HIV test kits and ARVs shortages. In addition, people in rural areas live very far from the clinic. So, when they come to a clinic to collect medication, and for follow-up, and being informed that there is no medication and HIV test kits, there is a possibility that people could get discouraged to come back to the clinic in future. The continuation of interrupted drug supplies would hinder the objective of achieving EMTCT in SA (Phaswana-Mafuya & Kayongo 2008).

The other barrier that has a serious potential to discourage women from attending clinics is healthcare workers’ negative attitude. Healthcare workers’ frustrations could be caused by several factors, some of which could be being overworked because of staff shortages, poor working environment, shortages of HIV test kits, ARVs and other medical supplies, amongst others. Despite working under frustrating conditions, they should uphold professional ethics when engaging with clients at all times. It cannot be right that women are made to feel disrespected and embarrassed when attending clinics. The frustrations of healthcare workers need to be addressed appropriately by the healthcare facility managers, which, in turn, would boost their morale and prevent potential loss to follow-up (LTFU) of women and children.

Over and above the healthcare workers’ negative attitude, some women reported that they sought ANC services later in their pregnancy because of various reasons. These include living far away from the clinic, transport availability and costs, fear to do HIV tests and disclosing results because of stigma, cultural beliefs and lack of support from their partners and family members (Fords et al. 2017).

Living far from a clinic means that there should be reliable and readily available transport. In other villages, transport is available, but women do not have money to pay for the transport in order to go to a clinic. Some women indicated having money for transport, but because of scarcity of transport in their villages, they could not go to the clinic. This means both groups were missing opportunities to benefit from the PMTCT services because of lack of transport and financial constraints. This finding concurs with other studies (Akal & Afework 2018; Ashaba et al. 2017; Frost et al. 2017; Rodriguez et al. 2017) which found that participants had financial and transport problems, and as a result did not go to clinic and some women gave birth at home. These situations should be avoided because there is a high risk of MTCT when women give birth at home without a healthcare worker, compared to those who deliver in the healthcare institution (Koye & Zekele 2013).

At times, pregnant women and their unborn babies die while waiting for transport to take them to the clinic. The roads in rural areas must be well developed and ambulance response time should be improved. Mobile clinics should have access to everyone regardless of geographic or weather conditions. Clinics should have suitable cars available to be utilised for home care visits and home deliveries, and these cars should be suitable to drive on the roads in rural areas.

Finally, cultural beliefs and stigma were found to be one of the significant barriers that hinder women and children from benefiting from the PMTCT programme. Women were scared to do HIV tests and disclose their HIV status to their partners or families because of the fear of stigmatisation and rejection. This has a further negative effect of non-adherence to ARVs (Tshililo et al. 2019). Some women resort to extreme measures, such as deciding not to attend ANC at all to escape having to do an HIV test because of the fear of stigma. In their research, Yah and Tambo (2018), Akal and Afework (2018), Fondoh and Mom (2017) and Cataldo et al. (2017) found that fear of stigma, discrimination and rejection, divorce, physical abuse and violence were preventing a majority of women in PMTCT programmes to disclose their HIV status to their partners or families. This behaviour shows that stigma continues to threaten the success of managing HIV-exposed children under five in rural communities. It is crucial for women to have support from their partners and families in order to disclose their HIV status without fear of being judged or accused of infidelity (Rodriguez et al. 2017). Oftentimes, once a child is born, the mother will leave her child at home with the grandmother and go back to the city for employment reasons. The grandmother or caregiver will look after a high-risk child (HIV-exposed) without knowing because the mother of a child has never disclosed her HIV status; which has a potential to disadvantage the child when comes to the care that the child should receive at the healthcare facilities. Disclosure of HIV status should be encouraged particularly for the immediate family members for better continuation of the management of HIV-exposed children who live with their grandmothers or caregivers.

Cultural beliefs should be revisited and be clearly explained where there is a potential danger to one’s life with regard to mixing traditional and western medicines. Traditional leaders and communities in rural areas should be engaged continuously on some of the cultural practices that appear to put the lives of the women and children at risk during pregnancy and those living with HIV. Doing so will steer the rural health community in the right direction to work towards the SDG motto of ‘leaving no one behind’ and achieving EMTC by 2030. In this review, the researchers found that some women were only performing cultural activities during their pregnancy, whereas others continued to use traditional remedies together with ART during pregnancy. A research conducted by Igwegbe, Ugboaja and Nwajiaku (2010) reported similar findings in that women were stopping the medication to explore a traditional healer’s treatment. A majority of the elderly population live in rural areas, and they are influential to their children (women) and grandchildren despite what HIV and AIDS education women are taught by healthcare workers in the clinic. They feel obliged to follow the instructions given by the elderly at home, hence taking ARVs and traditional medicine at the same time. Therefore, more work still needs to be performed in rural areas to neutralise deep-rooted cultural beliefs and practices in order to be flexible and adapt to current methods (Rodriguez et al. 2017).

## Limitations

This integrative literature review had the following limitations: it included only qualitative and quantitative peer-reviewed studies; it included only the studies that were conducted in rural areas in SA. Therefore, the findings of this study cannot be generalised across SA.

## Recommendations

The context-based intervention strategies to improve management of HIV-exposed children under 5 years are urgently needed.

There must be adequate highly skilled trained healthcare workers and they ought to be distributed to the primary healthcare level. Strategies to retain healthcare workers in rural healthcare facilities must be prioritised.

## Conclusion

The findings of this integrative literature review indicate the barriers that are threatening efficient management of HIV-exposed children in rural areas. The findings show that these bottlenecks should be addressed and corrected. The elimination of MTCT could be achieved only if there is a clear understanding regarding the barriers that exist on the ground level. Once the barriers are well understood, it is possible to design a context-based intervention strategy that would improve the management of HIV-exposed children under 5 years through the implementation of PMTCT policy guidelines in rural areas. The possible benefits that HIV-exposed children would enjoy as a result of having a specific, context-based intervention strategy that would seal the gaps, barriers that exist in the PMTCT and subsequently improve implementation of PMTCT guidelines cannot be overemphasised.

## References

[CIT0001] Adeniyi, V.O., Thomson, E., Ter Goon, D. & Ajayi, I.A., 2015, ‘Disclosure, stigma of HIV positive child and access to early infant diagnosis in the rural communities of OR Tambo district, South Africa: A qualitative exploration of maternal perspective’, *BMC Paediatrics* 15(1), 98. 10.1186/s12887-015-0414-8 PMC4549931 26306387

[CIT0002] Ajewole, O.J., Sparks, B.L. & Omole, O.B., 2013, ‘Uptake and factors that affect enrolment into the prevention of mother-to-child transmission of human immunodeficiency virus programme in rural Limpopo province’, *South African Family Practice* 55(6), 555–560. 10.1080/20786204.2013.10874416

[CIT0003] Akal, C.G. & Afework, D.T., 2018, ‘Status of prevention of mother-to-child transmission (PMTCT) services utilization and factors affecting PMTCT service uptake by pregnant women attending antenatal care clinic in selected health facilities of afar regional state, Ethiopia’, *Journal of Environmental and Public Health* 2018, Article ID 5127090, 7 pages. 10.1155/2018/5127090 PMC6311780 30651741

[CIT0004] Akinsanya, O.S., Wiseberg-Firtell, J.A., Akpomiemie, G., Adeniyi, O.V. & Kaswa, R.P., 2017, ‘Evaluation of the prevention of mother-to-child transmission programme at a primary health care centre in South Africa’, *South African Family Practice* 59(2), 56–60. 10.1080/20786190.2016.1254933

[CIT0005] Ashaba, S., Kaida, A., Coleman, J.N., Burns, B.F., Dunkley, E., O’Neil, K. et al., 2017, ‘Psychosocial challenges facing women living with HIV during the perinatal period in rural Uganda’, *PLoS One* 12(5), 0176256. 10.1371/journal.pone.0176256 PMC5411062 28459866

[CIT0006] Atkinson, D., 2014, *Rural-urban linkages: South Africa case study*, Working Paper Series 125, Latin American Centre for Rural Development, Santiago.

[CIT0007] Barron, P., Pillay, Y., Doherty, T., Sherman, G., Jackson, D., Bhardwaj, S. et al., 2013, ‘Eliminating mother-to-child HIV transmission in South Africa’, *Bulletin of the World Health Organization* 91, 70–74. 10.2471/BLT.12.106807 23397353 PMC3537246

[CIT0008] Bowling, A., 2009, *Research methods in health: Investigating health and health services*, Open University Press, Maidenhead.

[CIT0009] Cataldo, F., Chiwaula, L., Nkhata, M., Van Lettow, M., Kasende, F., Rosenberg, N.E. et al., 2017, ‘Exploring the experiences of women and health care workers in the context of PMTCT option B plus in Malawi’, *Journal of Acquired Immune Deficiency Syndromes (1999)* 74(5), 517. 10.1097/QAI.0000000000001273 28045712 PMC5340586

[CIT0010] Doherty, T., Chopra, M., Nsibande, D. & Mngoma, D., 2009, ‘Improving the coverage of the PMTCT programme through a participatory quality improvement intervention in South Africa’, *BMC Public Health* 9(1), 406. 10.1186/1471-2458-9-406 19891775 PMC2777166

[CIT0011] Ebonwu, J., Mumbauer, A., Uys, M., Wainberg, M.L. & Medina-Marino, A., 2018, ‘Determinants of late antenatal care presentation in rural and peri-urban communities in South Africa: A cross-sectional study’, *PLoS One* 13(3), 0191903. 10.1371/journal.pone.0191903 PMC5843210 29518082

[CIT0012] Egbujie, B.A., Grimwood, A., Mothibi-Wabafor, E.C., Fatti, G., Tshabalala, A., Allie, S. et al., 2018, ‘Impact of “Ideal Clinic” implementation on patient waiting time in primary healthcare clinics in KwaZulu-Natal province, South Africa: A before-and-after evaluation’, *South African Medical Journal* 108(4), 311–318. 10.7196/SAMJ.2017.v108i4.12583 29629682

[CIT0013] Fondoh, V.N. & Mom, N.A., 2017, ‘Mother-to-child transmission of HIV and its predictors among HIV-exposed infants at Bamenda regional hospital, Cameroon’, *African Journal of Laboratory Medicine* 6(1), 1–7. 10.4102/ajlm.v6i1.589 PMC5803518 29435421

[CIT0014] Fords, G.M., Crowley, T. & Van der Merwe, Anita S., 2017, ‘The lived experiences of rural women diagnosed with the human immunodeficiency virus in the antenatal period’, *SAHARA-J: Journal of Social Aspects of HIV/AIDS* 14(1), 85–92. 10.1080/17290376.2017.1379430 PMC5639609 28949277

[CIT0015] Frost, L., Jenkins, L.S. & Emmink, B., 2017, ‘Improving access to health care in a rural regional hospital in South Africa: Why do patients miss their appointments?’, *African Journal of Primary Health Care & Family Medicine* 9(1), 1–5. 10.4102/phcfm.v9i1.1255 PMC5387369 28397521

[CIT0016] Goga, A., Chirinda, W., Ngandu, N.K., Ngoma, K., Bhardwaj, S., Feucht, U. et al., 2018, ‘Closing the gaps to eliminate mother-to-child transmission (MTCT) of HIV in South Africa: Understanding MTCT case rates, factors that hinder the monitoring and attainment of targets, and potential game changers’, *Southern African Medical Journal* 108(3 Suppl 1), S17–S24. 10.7196/SAMJ.2017.v108i3b.12817

[CIT0017] Habedi, D., Nolte, A. & Temane, M.A., 2016, ‘Factors affecting the availability of the prevention of mother to child transmission of HIV programme at rural health care facilities of Madibeng sub-district’, *Africa Journal of Nursing and Midwifery* 18(1), 90–102. 10.25159/2520-5293/397

[CIT0018] Hanrahan, B.A. & Williams, A., 2017, ‘Prevention of mother-to-child transmission of HIV guidelines: Nurses’ views at four primary healthcare facilities in the Limpopo province’, *Southern African Journal of HIV Medicine* 18(1), a690. 10.4102/sajhivmed.v18i1.690 PMC5843237 29568626

[CIT0019] Igwegbe, A.O., Ugboaja, J.O. & Nwajiaku, L.A., 2010, ‘Prevalence and determinants of non-adherence to antiretroviral therapy among HIV-positive pregnant women in Newi, Nigeria’, *International Journal of Medicine and Medical Sciences* 2(8), 238–245.

[CIT0020] Kaswa, R., Rupesinghe, G.F. & Longo-Mbenza, B., 2018, ‘Exploring the pregnant women’s perspective of late booking of antenatal care services at Mbekweni health centre in Eastern Cape, South Africa’, *African Journal of Primary Health Care & Family Medicine* 10(1), 1–9. 10.4102/phcfm.v10i1.1300 PMC6111382 30035599

[CIT0021] Kendall, C., Claessens, L., Dorward, J., Mfeka, G. & Gate, K., 2015, ‘Reasons for failure of prevention of mother-to-child HIV transmission in a rural South African district hospital’, *Southern African Journal of HIV Medicine* 16(1), a365. 10.4102/sajhivmed.v16i1.365 PMC5843254 29568588

[CIT0022] Koye, D.N. & Zeleke, B.M., 2013, ‘Mother-to-child transmission of HIV and its predictors among HIV-exposed infants at a PMTCT clinic in northwest Ethiopia’, *BMC Public Health* 13(1), 398. 10.1186/1471-2458-13-398 23621862 PMC3639796

[CIT0023] Mafune, R.V., Lebese, R.T. & Nemathaga, L.H., 2017, ‘Challenges faced by caregivers of children on antiretroviral therapy at Mutale municipality selected healthcare facilities, Vhembe district, Limpopo province’, *Curationis* 40(1), 1–9. 10.4102/curationis.v40i1.1541 PMC6091811 28893073

[CIT0024] Massyn, N., Pillay, Y., Padarath, A. (eds.), 2018/19, *District Health Barometer*, Health System Trust, Durban.

[CIT0025] Meehan, S., Leon, N., Naidoo, P., Jennings, K., Burger, R. & Beyers, N., 2015, ‘Availability and acceptability of HIV counselling and testing services. A qualitative study comparing clients’ experiences of accessing HIV testing at public sector primary health care facilities or non-governmental mobile services in Cape Town, South Africa’, *BMC Public Health* 15(1), 845. 10.1186/s12889-015-2173-8 26329262 PMC4557635

[CIT0026] Mlambo, M., Penn, C., Peltzer, K. & Phaswana-Mafuya, N., 2018, ‘Multiple perspectives on factors affecting early antenatal care attendance in the context of PMTCT in a rural district of South Africa’, *Gender and Behaviour* 16(1), 10646–10667.

[CIT0027] National Department of Health, 2014, *National consolidated guidelines for the prevention of mother-to-child transmission of HIV (PMTCT) and the management of HIV in children, adolescents’ and adults*, National Department of Health, Pretoria, viewed 17 April 2019, from http://www.kznhealth.gov.za/family/HIV-Guidelines-Jan2015.pdf.

[CIT0028] Nkomo, P., Davies, N., Sherman, G., Bhardwaj, S., Ramokolo, V., Ngandu, N.K. et al., 2015, ‘How ready are our health systems to implement prevention of mother to child transmission option B?’, *Southern African Journal of HIV Medicine* 16(1), 1–5. 10.4102/sajhivmed.v16i1.386 PMC5843177 29568595

[CIT0029] Pearson, A., 2004, ‘Balancing the evidence: Incorporating the synthesis of qualitative data into systematic reviews’, *JBI Reports* 2(2), 45–64. 10.1111/j.1479-6988.2004.00008.x

[CIT0030] Peltzer, K., Prado, G., Horigian, V., Weiss, S., Cook, R., Sifunda, S. et al., 2016, ‘Prevention of mother-to-child transmission (PMTCT) implementation in rural community health centres in Mpumalanga province, South Africa’, *Journal of Psychology in Africa* 26(5), 415–418. 10.1080/14330237.2016.1219537 28286412 PMC5340272

[CIT0031] Phaswana-Mafuya, N. & Kayongo, D., 2008, ‘Barriers to implementation of PMTCT program in the Eastern Cape of South Africa’, *Current Politics and Economics of Africa* 1(2), 161–181.

[CIT0032] Pitt, V., Powis, D., Levett-Jones, T. & Hunter, S., 2012, ‘Factors influencing nursing students’ academic and clinical performance and attrition: An integrative literature review’, *Nurse Education Today* 32(8), 903–913. 10.1016/j.nedt.2012.04.011 22595612

[CIT0033] Rodriguez, V.J., LaCabe, R.P., Privette, C.K., Douglass, K.M., Peltzer, K., Matseke, G. et al., 2017, ‘The Achilles’ heel of prevention to mother-to-child transmission of HIV: Protocol implementation, uptake, and sustainability’, *SAHARA-J: Journal of Social Aspects of HIV/AIDS* 14(1), 38–52. 10.1080/17290376.2017.1375425 PMC5638135 28922974

[CIT0034] Sandelowski, M., 2000, ‘Combining qualitative and quantitative sampling, data collection, and analysis techniques in mixed method studies’, *Research in Nursing & Health* 23(3), 246–255. 10.1002/1098-240X(200006)23:3<246::AID-NUR9>3.0.CO;2-H 10871540

[CIT0035] Sandelowski, M., Voils, C.I. & Barroso, J., 2006, ‘Defining and designing mixed research synthesis studies’, *Research in the Schools* 13(1), 29. 20098638 PMC2809982

[CIT0036] Sewell, S.J. & Desai, S., 2016, ‘The impacts of undeveloped roads on the livelihoods of rural women’, *Review of Social Sciences* 1(8), 15–29. 10.18533/rss.v1i8.40

[CIT0037] Sithole, P.M. & Khunou, S.H., 2016, ‘Factors contributing to missed opportunities in the prevention of mother to child transmission programme in the sub-district of Ngaka Modiri Molema, North West province, South Africa’, *African Journal for Physical Activity and Health Sciences (AJPHES)* 22(31), 667–678.

[CIT0038] Skinner, D., Mfecane, S., Gumede, T., Henda, N. & Davids, A., 2005, ‘Barriers to accessing PMTCT services in a rural area of South Africa’, *African Journal of AIDS Research* 4(2), 115–123. 10.2989/16085900509490350 25870888

[CIT0039] Sprague, C., Chersich, M.F. & Black, V., 2011, ‘Health system weaknesses constrain access to PMTCT and maternal HIV services in South Africa: A qualitative enquiry’, *AIDS Research and Therapy* 8(1), 10. 10.1186/1742-6405-8-10 21371301 PMC3058008

[CIT0040] Statistic South Africa, 2018, *Mid-year population estimates, 23 July 2018*, Statistic South Africa, Pretoria.

[CIT0041] Suryavanshi, N., Mave, V., Kadam, A., Kanade, S., Sivalenka, S., Kumar, V.S. et al., 2018, ‘Challenges and opportunities for outreach workers in the prevention of mother to child transmission of HIV (PMTCT) program in India’, *PLoS One* 13(9), 0203425. 10.1371/journal.pone.0203425 PMC6122806 30180186

[CIT0042] Torraco, R.J., 2016, ‘Writing integrative literature reviews: Using the past and present to explore the future’, *Human Resource Development Review* 15(4), 404–428. 10.1177/1534484316671606

[CIT0043] Tshililo, A.R., Mangena-Netshikweta, L., Nemathaga, L.H. & Maluleke, M., 2019, ‘Challenges of primary healthcare nurses regarding the integration of HIV and AIDS services into primary healthcare in Vhembe district of Limpopo province, South Africa’, *Curationis* 42(1), 1–6. 10.4102/curationis.v42i1.1849 PMC6417489 30843402

[CIT0044] United Nations, 2016, *Sustainable Development Goals Report*, United Nations, Geneva.

[CIT0045] Useh, U., Keikepe, A., Montshiwagae, B., Mothoagae, R. & Senna, D., 2013, ‘Knowledge and attitude of pregnant women towards mother to child transmission (MTCT) of HIV and AIDS in a local clinic in Mafikeng, South Africa’, *Studies on Ethno-Medicine* 7(3), 163–169. 10.1080/09735070.2013.11886457

[CIT0046] Whittemore, R. & Knafl, K., 2005, ‘The integrative review: Updated methodology’, *Journal of Advanced Nursing* 52(5), 546–553. 10.1111/j.1365-2648.2005.03621.x 16268861

[CIT0047] Wilford, A., Phakathi, S., Haskins, L., Jama, N.A., Mntambo, N. & Horwood, C., 2018, ‘Exploring the care provided to mothers and children by community health workers in South Africa: Missed opportunities to provide comprehensive care’, *BMC Public Health* 18(1), 171. 10.1186/s12889-018-5056-y 29361926 PMC5781263

[CIT0048] Yah, C.S. & Tambo, E., 2018, ‘Why is mother to child transmission (MTCT) of HIV a continual threat to new-borns in sub-Saharan Africa (SSA)?’, *Journal of Infection and Public Health* 12(2), 213–223. 10.1016/j.jiph.2018.10.008 30415979

